# The High School Competencies Scale (H-Comp Scale): A First Validation Study

**DOI:** 10.3390/ejihpe11020041

**Published:** 2021-06-17

**Authors:** Diego Boerchi, Paola Magnano, Ernesto Lodi

**Affiliations:** 1 Department of Psychology, University Cattolica del Sacro Cuore, 20123 Milan, Italy; 2 Faculty of Human and Social Sciences, Kore University, 94100 Enna, Italy; paola.magnano@unikore.it; 3 Department of Humanities and Social Sciences, University of Sassari, 07100 Sassari, Italy; elodi@uniss.it

**Keywords:** academic performance, high school, self-efficacy, students’ competencies

## Abstract

Researchers widely explored non-intellective study factors because they play a central role in academic performance and are potentially more modifiable than intellective ones. The scientific literature suggests that the non-intellective factors can be classified into three main areas: self-concept, which refers to self-esteem and efficacy, motivation and emotional reactions; the area of study, related to study dedication and operative skills; and the area of relationships, comprising those with family, fellow students and teachers. Basing on these findings, the C-Comp Scale has been developed and tested in the past, addressed to college students. This study aimed to adapt and test a new version of this questionnaire on high school students. Methods. A pilot study was conducted on 364 Italian high school students to adapt and test the new version of the questionnaire, called the H-Comp Scale. The following study, conducted on 792 Italian high school students, provided further evidence of its reliability, structural validity, and concurrent validity with general self-efficacy, academic self-efficacy, social self-efficacy, and academic performance. Results. The H-Comp Scale showed to possess excellent reliability and structural and concurrent validity. The final version is composed of twelve subscales, aggregated in three areas, with just 48 items: Study (Intrinsic Motivation, Extrinsic Motivation, Time Management, Study Dedication), Self (Learning Assessment, General Self-Esteem, Self-Efficacy, Reaction to Failures, Emotional Control), and Relationships (Family Relationships, Fellow Student Relationships, Teacher Relationships). Conclusions. The H-Comp Scale would be a useful and easy-to-use instrument to support school counselors, tutors, teachers, and researchers in exploring different types of non-intellective variables, to better project educational intervention aimed to improve high school students’ academic performance and satisfaction.

The literature about the role of non-intellective factors in school and academic achievement is vast—the researchers have widely explored these factors because they are potentially more modifiable than the intellective ones. An in-depth analysis of the scientific literature and the instruments used in this field has suggested that the non-intellective factors can be classified into three main areas: self-concept, which refers to self-esteem and efficacy, motivation, and emotional reactions; the area of study, related to study dedication and operative skills; and the area of relationships, comprising those with family, fellow students, and teachers. On this theoretical base, we have developed the College Competences Scale (C-Comp Scale), a new instrument to assess the most represented non-intellective predictors of academic success. It has been validated on an Italian sample of university students and showed excellent psychometric properties in terms of reliability, factor structure, and validity [1,2]. The current study presents the adaptation of this questionnaire to high school students, given that the literature review suggests the same dimensions as predictors of academic success also with this target. This scale gives an innovative contribution as it offers the opportunity to evaluate many dimensions related to the students’ achievement using a unique instrument with an adequate number of items. Its concision makes the tool suitable also for the screening of risky underachievement students, conducted by psychologists, school counselors, and teachers, to help them to work on their positive school adjustment and to improve both academic performance and wellbeing. The study presented aims to explore the psychometric properties—reliability, validity, and factor structure—of the High School Competences Scale (H-Comp Scale), which is the adaptation of the C-Comp Scale to high school students.

## 1. Introduction

The factors that affect the quality of performance of students are numerous [3], which is why identifying the most relevant variables in the quality of academic performance is a very complex and challenging job. The review of the existent literature previously conducted for the construction of the C-Comp Scale [1] highlighted that the most relevant studies in classifying the non-intellective factors predicting academic performance are by Richardson and colleagues’ [4] and Goodman and colleagues’ [5]. Both publications underlined the complexity of the issue and classified the predictors in (a) personality traits or dimensions, including self-esteem and self-efficacy; (b) motivational factors; (c) self-regulatory abilities; and (d) psychosocial and relational factors.

The self-determination theory [6] offers a consistent theoretical framework to the C-Comp Scale and, subsequently, the H-Comp Scale. The three areas of the C-Comp Scale seem to respond to the importance of satisfying the three psychological needs essential for psychological growth, integrity, and wellbeing. Specifically, the self-concept area promotes an adequate response to the need for autonomy that implies that the perception of the causality of individuals’ own actions is put in internal factors, rather than in pressures coming from the external environment. The area of study promotes an adequate response to the need for competence, which leads individuals to cope with challenges, producing an optimal level of stimulation in a valued context, such as an educational environment. The area of relationships is related to the need for feeling connected to others, which implies a sense of belonging to a community, group, or culture. If individuals can satisfy the three psychological needs in their context, they will be more proactive to cope with the internal and external challenges towards their optimal development, growth and functioning in their environment. Therefore, several studies showed that self-determined behavior is related to many life outcomes and to the quality of life, underlining the importance to support the development of self-determination in different contexts (e.g., educational and work environment) to facilitate the achievement of educational and work goals [7,8]. 

**Self-esteem**. Regarding the role of self-esteem in academic achievement, the research is not definitive, showing the different types of results depending on the cross-sectional or longitudinal nature of the studies [9]. As underlined by El-Anzi [10], different researchers have demonstrated that academic achievement and self-esteem are positively correlated [11], even though the results did not determine the direction of the relationship. **Self-efficacy**. Self-efficacy, unlike self-esteem, has a clear relationship with academic achievement and is the strongest predictor of performance in many domains [12]. In the literature review reported by Caprara, Vecchione, Alessandri, Gerbino, and Barbaranelli [13], different studies have demonstrated that self-efficacy beliefs are significant predictors of academic achievement. As reported by the authors, students’ academic self-efficacy beliefs were significant antecedents of students’ academic continuance and achievement, college performance, persistence, and GPA [14]. The role of self-esteem and self-efficacy in academic achievement can be read in light of the framework of self-determination theory, as promoting the satisfaction of the need for autonomy and self-growth.

**Extrinsic and intrinsic motivation**. A number of studies have demonstrated that motivation has significant influences on various academic outcomes [15], such as academic achievement [16], intention to drop out [17], and absenteeism [18]. We agree with Ayub [19] that motivation is a significant factor for academic learning and achievement from childhood to adolescence [20] and is related to various outcomes such as curiosity, persistence, learning, and performance [6]. As stated by Guay, Vallerand, and Blanchard [21], intrinsic motivation refers to carrying out an activity for itself, experiencing pleasure and satisfaction inherent in the activity; extrinsic motivation, on the other side, is related to a wide variety of behaviors where the goals of action extend beyond those inherent in the activity itself [22]. However, according to the self-determination theory, different types of extrinsic motivations have been proposed: from lower to higher levels of self-determination, external and identified regulations can be distinguished. External regulation occurs when behavior is regulated by rewards or to avoid negative consequences and is characterized by the individual experiences an obligation to behave in a specific way; identified regulation occurs when the activity is not performed for itself but as a means to an end.

**Self-regulatory abilities: reaction to failures and emotional control**. Reaction to failure and emotional control can be included among the self-regulatory abilities, that in a social cognitive framework—as previously found [1]—comprise (a) setting specific goals; (b) using task strategies such as elaborating, organizing, and rehearsing; (c) having high levels of self-efficacy and intrinsic interest; and (d) self-monitoring and self-reflecting on performance outcomes [22,23]. Reaction to failure and emotional control pertains to the last point. Students with less fear of failure consider failure situations as a learning opportunity rather than an indictment of self-worth, avoiding harsh self-condemnation; this ability is associated with higher levels of perceived competence [24]. In previous research, the relationship between the tendency to react in a negative way and low levels of self-efficacy has been demonstrated [25], prompting the idea that individuals who are indulgent toward their weaknesses, avoid over-identifying with their emotional reactions, or judge themselves too severely should develop more positive perceptions of their abilities [24]. Emotional control can be considered the other side of test anxiety and is referred to as the difficulty in managing exams with professors [1]. It is a cognitive, emotional, physiological, and behavioral state of activation that happens to anticipate a potentially harmful outcome, specifically in situations that require a formal evaluation [26]. Beyond the well-known u-inverted relationship between anxiety and performance, many studies have focused on the relationship between test anxiety and academic performance at every educational level, finding similar results: Cassady and Johnson [27] highlighted that higher levels of test anxiety are related to lower levels of academic achievement, problem-solving, memory, and grades; Chapell, Blanding, Silverstein, and colleagues [28] confirmed that test anxiety is linked to reductions in GPA both in undergraduate and graduate students. Additionally, Bembenutty [26] showed that high levels of test anxiety were negatively associated with self-efficacy and were related to less adaptive task values and less frequent use of cognitive learning strategies. Other studies come to similar conclusions, finding that test anxiety was a predictor of poor performance and poor test-taking [29].

**Self-regulatory learning strategies: time management, study dedication, and learning assessment.** As previously underlined, in the framework of self-determination theory, the area of study promotes an adequate response to the need for competence. The effective use of the regulation of study time and one’s surrounding environment is included in academic self-regulation; this ability allows us to accomplish learning goals [30]. Following the study of Richardson and colleagues [4], time management is defined as the capacity to self-regulate study time and activities. Kitsantas et al. [22] underlined that managing time effectively and staying in settings that favor learning is positively related to better performance [31] and good adjustment in a school context. In general, according to various research, time management and organizational strategies have been found to be predictors of academic achievement [32] and retention [33] in school and college. Study dedication is defined as the amount of time spent on homework and class attendance [1] and has been found to have a positive effect on student learning and academic performance [34]. In a metanalytic review, Richardson et al. [4] included the effort regulation in the behavioral self-regulatory capacities [35] that comprises self-management of motivation or persistence when faced with challenging academic situation. The literature review reported by Carbonaro [36] substantiates the same results: students’ effort, despite the different labels used across studies, is related to higher achievement. Another important dimension comprised of self-regulation is the students’ ability to self-evaluate their learning before the exam accurately. The results of Zulkiply’s [37] research showed a positive relationship between metacognition and students’ academic performance: students who do well in the examination have higher scores in metacognition measures. Students who have metacognition awareness are able to plan their study activity, monitor their learning, evaluate their study strategies and the outcomes of their learning activity, estimating their strengths and their weaknesses realistically. These results confirm those found in previous studies: metacognitive learners are more strategic and tend to be successful learners [38].

**Relational factors.** In addition to individual factors, contextual and environmental aspects also play an important role in students’ academic success. Referring to the self-determination theory, this area is related to the need for connectedness. Farooq, Chaudhry, Shafiq, and Berhanu [39] underlined that teachers, peers, and families are an important source of aid and assistance to students, favoring the improvement of their academic performance. This social support is pivotal in the achievement of performance goals for the students [40]. Among the contextual aspects, the literature review suggested that the relationships that the students have with their parents, teachers, and peers seem particularly significant in affecting academic achievement [1]. In a very general sense, Castro and colleagues [41] consider parental involvement as the active participation of parents in all aspects of their children’s social, emotional, and academic development, including the support in academic choices [42]. More specifically, the expression “parental involvement” is used to indicate (1) the expectations that parents have about their children’s academic future, (2) the control they have upon homework, or the degree of involvement in helping their children in the homework, or (3) how frequently the parents go to school to talk with the teachers. According to the definition given to parental involvement, the pertinent literature has shown different results in the relationship with academic outcomes. In fact, Wilder [43], in her meta-synthesis study, underlined that beyond the strong positive relationship between the two constructs regardless of the definition of parental involvement, when parental involvement was defined as parental expectations for academic achievement of their children, this relationship was the strongest. Castro et al. [41] revised a great number of meta-analytic studies conducted in the last twenty years, summarizing as follows: “On the one hand, we have those of a more general nature that study the overall relation between spontaneous parent participation and academic achievement […] that studied the effect of parental involvement in different ethnic groups. On the other hand, we have the meta-analysis based on the evaluation of programs of parent participation […] The reviews show a significant, although moderate, association between parent participation and children’s academic results” (p. 35). The findings of their study, finally—consistently with the previous meta-analytical literature—showed that when parents have high academic expectations for their children and develop and maintain communication with them about school activities and schoolwork, there are the strongest associations between type of parental involvement and academic achievement. As well as the role of parents, the role of teachers’ support in students’ academic outcomes has also been largely demonstrated in many different studies. Recently, Longobardi, Prino, Marengo, and Settanni [44] found that a student’s experience of positive relationships with the teachers develops a greater interest in school activities, motivating and willing them to learn [45]; these students show, in turn, higher academic achievement [46]. Moreover, the quality of relationships with peers is the third relational factor affecting students’ academic achievement. Wentzel’s studies (e.g., [47]) have demonstrated that children who enjoy positive relationships with their peers also tend to be more engaged in and even to excel at academic tasks more than those who experience problems with peers. A relationship between the quality of peer relations and both students’ academic orientations and their school performance has been found [48].

**In the following part of the paper, two studies are presented:** The Pilot study, aimed to adapt and test the C-Comp Scale on high school students, and the Main study, aimed to seek further evidence of the validity and reliability of the H-Comp Scale.

**Data Analysis**. Before proceeding with the description of the studies, here we describe the analyses we have conducted. Confirmatory factor analyses (CFA) were tested using the maximum likelihood method and AMOS software. Goodness-of-fit indices were examined through the chi-square test, root mean square error of approximation (RMSEA), and comparative fit index (CFI). A non-significant chi-square is desired, which would suggest that the observed and reproduced covariance matrix does not significantly differ, but models with a large sample can only be evaluated by RMSEA and CFI because this test is sensitive to sample size. Models with acceptable fit also presented RMSEA < 0.08 and CFI > 0.90 [49], whereas models with optimum fit presented RMSEA < 0.05 and CFI > 0.95 [50].

Measurement invariance of the models was tested through multigroup CFAs. Different levels of invariances were tested [51]: configural invariance (the overall model fit well in both groups), metric invariance (the factor loadings were constrained to be equal in male and female groups), strong invariance (the factor loading and intercept were constrained to be equal in male and female groups), and strict invariance (the factor loadings, intercept and residuals were constrained to be equal in male and female groups). Models were compared step by step, comparing the more constrained model with the previous, less constrained one. Fit indices were compared to state that two models were not different, Δχ^2^ should not be significant, and ΔCFI and ΔRMSEA should be lower than 0.010 and 0.015 [52], respectively. Because of the sensitiveness of χ^2^ to sample size [53], we considered that two models were not different also when Δχ^2^ is significant, but ΔCFI and ΔRMSEA are lower than the cut-off.

**Pilot study**

## 2. Materials and Methods

**Aims.** This study aimed to adapt and test the C-Comp Scale on high school students and to test the basic structure of the H-Comp Scale by:

1. **Testing the factorial structure of the three areas which compose the scale through confirmative factorial analysis (CFA);** providing evidence regarding the internal consistency through Cronbach’s alpha.

**Scale adaptation**. In the first step, the authors adapted the scale autonomously, and some items were modified to reach a complete agreement. Following, we administered the scale to a group of 21 high school students with the aim of testing if all the items were understandable and with adequate variance in the answers. After the administration, a focus group gave some suggestions to improve some of the items and completely change one of them.

As for the C-Comp Scale, the H-Comp Scale is composed of 48 items, divided into 12 subscales grouped into three macro-areas:

1. Area of Study: Intrinsic Motivation (i.e., *Generally, I study willingly because I like doing it*), Extrinsic Motivation (i.e., *I always find a way to study, even when I am not very interested*), Time Management (i.e., *I can plan my study workload so that I am not late*), Study Dedication (i.e., *I study with perseverance*);
2. Area of Self: Reaction to Failures (i.e., *I do not get discouraged when I face difficulties in my studies*), Learning Assessment (i.e., *I can evaluate with some accuracy if I am ready or not for a written or oral school test*), Self-Efficacy (i.e., *I consider myself a student with good study skills*), Emotional Control (i.e., *I am not anxious when I take a written or oral school test*), General Self-Esteem (i.e., *I have good self-esteem*);
3. Area of Relations: Family Relationships (i.e., *I involve my family as much as possible in my studies*), Fellow Student Relationships (i.e., *When I need help, I ask my fellow students*), Teachers Relationships (i.e., *I have good relationships with all my teachers*).

A part the General Self-Esteem subscale, all the dimensions directly concern attitudes and behaviors related to the school activities and context.

**Participants**. The study was conducted on 364 Italian high school students, fairly equilibrated by gender (54% female and 46% male) with ages ranging from 14 to 19 years old, quite equally distributed among the five grades. They were attending three different lyceums (scientific, classic, and artistic) and different technical institutes in Italy. The students were reached partly through direct contact with three schools and partly as a convenience sample. In this way, we have covered adequate representativeness of different types of Italian high schools. It was not necessary to determine any exclusion criterion because both the content and the level of difficulty required to compile the questionnaire were congruent with the skills and motivation of students attending classes in high schools.

**Procedure**. Students answered the questionnaire in the paper-pencil version during school time. They were asked to compile the questionnaire anonymously, indicating how correct each sentence related to their school experience was using a 5-point Likert scale: (1) “not at all” (2), “a little” (3), “somewhat” (4), “very” (5), “completely”. The questionnaire was administered with the H-Sat Scale, which is a five-dimension questionnaire aimed to measure students’ scholastic satisfaction, whose validation has already been published in different articles [54,55]. The whole study was approved by the Internal Review Board of the Kore University of Enna, Italy.

## 3. Results of the Pilot Study

The distribution was normal for all the items, with skewness ranging from −0.470 to 0.647 and kurtosis ranging from −0.995 to 0.217. Means ranged from 2.35 to 3.73 and standard deviation (SD) from 0.824 to 1.351. The three areas’ structure was tested through CFA.

CFA confirmed the structure of the three models with good fit indices (Table 1). Standardized regression weights ranged from 0.503 to 0.868.

We tested reliability with Cronbach’s alpha, and all the subscales showed to possess good internal consistency.

**Main study**

## 4. Materials and Methods

Aims. The main study sought further evidence of the validity and reliability of the H-Comp Scale by:

(1) Testing the factorial structure of the three areas which compose the scale through confirmative factorial analysis (CFA);
(2) Providing evidence regarding the internal consistency through Cronbach’s alpha;
(3) Testing its concurrent validity with the Self area related with general self-efficacy, the Study area with academic self-efficacy, and the Relationships area with social self-efficacy;
(4) Testing its concurrent validity with academic performance.

**Participants.** A total of 792 Italian high school students participated in the main study. They were attending three different schools: linguistic lyceum, scientific lyceum, and technical institute. The schools were in Milan’s suburb, Italy, allowing us to reach a target which, from a socio-economical point of view, is located in a middle position between the center of the city, where families with higher resources and qualifications live, and villages. Participation in the study was proposed to all the students attending the courses from grade 9 to 13, specifying they were free not to participate and to give up at any time without providing any reason. Around 96% of the students returned the informed consent signed by their parents or by themselves if they of legal age. According to our sample size calculation, our sample is capable of reflecting the target population for a confidence level of 95% and at a 3.41% margin of error. One of the researchers, a psychologist, administered an online version of the questionnaire at school, one class at a time in April–May, so that also the freshmen may have gained sufficient experience in that school. The sample was not equilibrated by gender: males were more numerous in the scientific lyceum (75.6%) and technical institute (95.9%), whereas, in they linguistic lyceum, students were mainly females (84.2%). On the contrary, subsamples were homogeneous by age, with 98.2% ranging from 14 to 20 years old, quite equally distributed among the five grades. Researchers informed the students previously that they were free not to participate in the study, the compilation was nominal, and it would provide an individual profile. Because we guessed that some students were not sufficiently motivated to participate in the study, we used the Mahalanobis distance to find outliers in multivariate data considering all the subscales of the H-Comp Scale: 59 students (7.4% of the sample) were identified as outliers and no more considered in the study. The percentage was slightly higher in the technical institute subsample (11.1%) and lower in the other two subsamples (5.0% in linguistic lyceum and 4.3% in scientific lyceum).

**Procedure.** The psychologist presented the study to the students, class by class, explaining to them that it was aimed to assess their perception “about their experience as a high school student” and that, in the following weeks, they will be provided with a personal profile useful to understand the reasons of their school performance and satisfaction. Then, during school time, students divided by class were conducted in the computer classroom to fill out the online version of the questionnaire. As for the pilot study, the H-Comp Scale was administered after the H-Sat Scale. In this study, students also compiled the questionnaires My Life as a Student [56] and the Satisfaction with Life Scale [57], which will not be considered in this article, and the General Self-Efficacy Scale [58], the Scholastic Perceived Self-Efficacy and the Social Perceived Self-Efficacy [59], and some questions on demographic data (sex, age, course attended and class).

**Measures.** H-Comp Scale. The questionnaire was the same as that used in the pilot study. Descriptive statistics showed that both items and subscales were normally distributed (see Table 2 for subscales’ details and Table S1 in Supplementary Materials for the list of the items in English with their psychometrics).

When comparing males and females, we found strict measures of invariance in all the models considering ΔCFI and ΔRMSEA (Table 3, Table 4 and Table 5). 

In addition, comparing the subsamples of students attending different grades, which ranged from Grade 9 to Grade 13, we found strict measures of invariance in all the models excepted the strict invariance for the Self and Relationships models (Table 6, Table 7 and Table 8).

GSES—General Self-Efficacy Scale [58] using the Italian adaptation [59]. It is a self-reported, one-dimensional scale on general self-efficacy, composed of 10 items on a 5-point Likert scale. The internal consistency index was optimum (α = 0.916).

ASCP—Scholastic Perceived Self-Efficacy [60]. It is a one-dimensional scale composed of five items, on a 5-point Likert scale, on self-efficacy about some specific topics such as math, science, history, and geography, and twelve items that refer to a wide range of behaviors connected to school such as homework, study commitment, and study planning. The internal consistency index was optimum (α = 0.870).

ASPG—Social Perceived Self-Efficacy [60]. It is a one-dimensional scale composed of 13 items, on a 5-point Likert scale, on different skills useful to build good relationships with classmates and other peers. The internal consistency index was optimum (α = 0.881).

SP—Scholastic performance. It consists of the mean of the students’ marks in the different courses they attended. Considering that marks can range from 0 to 10 and that 6 is the minimum required to pass the course, our sample had a mean of 6.159 (SD = 0.904), and students’ scores were normally distributed (skewness = −0.027; kurtosis = 0.149).

## 5. Results of the Main Study

**H-Comp Scale Structural Validity**. We tested three models previously described through CFA using the maximum likelihood method. Table 9 shows the goodness-of-fit -indices that were good or acceptable for all the models.

For the Study model, standardized regression weights ranged from 0.455 to 0.818, standard errors from 0.039 to 0.112, and the correlations between the four scales were all statistically significative with *p* < 0.001 and ranged from 0.611 to 0.804.

For the Self model, standardized regression weights ranged from 0.500 to 0.807, standard errors from 0.046 to 0.092, and the correlations between the four scales were all statistically significative with *p* < 0.001 and ranged from 0.271 to 0.546, showing to be a moderated part of the correlation between Self-Efficacy and Learning Assessment, which was 0.711.

For the Relationship model, standardized regression weights ranged from 0.484 to 0.813, standard errors from 0.048 to 0.079, and the correlations between the four scales were all statistically significative with *p* < 0.001 and ranged from 0.312 to 0.327.

The Relationship model showed the most stable in the population, having the expected cross validation index (ECVI) for the saturated model of 0.213, while for both the Study and Self models, it was 0.372.

**H-Comp Scale Reliability**. As shown in Table 10, the majority of the twelve subscales had good internal consistency. Just the learning assessment was slightly lower of 0.700: after controlling the behavior of each item of the subscale, it was clear that this index was not due to the weakness of a specific one.

**H-Comp Scale Concurrent Validity with Self-Efficacy**. We hypothesized that high school competencies were related to different types of self-efficacy. Table 5 shows that the four subscales of the Study area correlated mainly with scholastic self-efficacy; the five subscales of the Self area correlated mostly with the general self-efficacy; parents’ and teachers’ relationships subscales correlated mostly with scholastic self-efficacy, while the fellow students’ relationships subscale correlated mostly with the social-friendly self-efficacy. The other subscales correlated with social self-efficacy, but more moderately.

**H-Comp Scale Concurrent Validity with Scholastic Performance**. We hypothesized that high school competencies were related to scholastic performance. Table 11 shows that study dedication and study self-efficacy were the subscales more correlated with the mean of the students’ marks in the different courses they attended, followed by extrinsic motivation and time management. Intrinsic motivation and relationships with teachers were moderately correlated, while the reaction to failures and emotional control were the only two that did not correlate with scholastic performance.

## 6. Discussion

The High School Competencies Scale showed to possess good psychometric properties in terms of reliability and validity, similar to those of the original scale designed and tested with college students. The questionnaire evaluates behaviors, attitudes, and students’ role perceptions that can affect scholastic performance. It consists of 48 items, four for each of the 12 subscales, which are grouped into three areas: Study (Intrinsic Motivation, Extrinsic Motivation, Time Management, and Study Dedication); Self (Reactions to Failures, Learning Assessment, Self-Efficacy, Emotional Control, and General Self-Esteem); and Relationships (Family Relationships, Fellow Student Relationships, and Teacher Relationships).

Non-intellectual competences affect students’ school careers at different educational levels. A large number of studies, both at high school and college educational levels, showed the incidence of non-cognitive variables on academic performance (i.e., [61]). As we hypothesized, almost all the subscales of the H-Comp Scale correlated with school performance, confirming both the literature and the results we obtained with the college students [1].

The Study area seems to be the most crucial since it contains three of four subscales more related to the school performance. The study dedication was the non-intellective competence more related to the performance; coherently with previous studies (i.e., [14,21,33]), students who dedicate sincere effort and spend a significant amount of time in study, who are more motivated and able to manage their time, are more likely to obtain better performance and achieve their academic goals. 

The results of the Self area, coherently with a large body of studies, confirmed that students who report a higher level of confidence in their study skills are those with a better performance in their academic career. Socio-cognitive theory and different studies highlighted the strong relationship between self-efficacy beliefs and academic performance [12,13,62] and also between self-efficacy and domain-specific satisfaction, such as academic satisfaction [2,63]. The low relationship between performance and self-esteem could be considered unexpected, but this result can be explained by the nature of self-esteem, which is a more general construct, different from self-efficacy perceptions which are more specific, and context-related. The previous scientific literature on this topic is controversial. For example, recent studies indicated that academic achievement affects self-esteem among younger students but not in eighth and ninth-grade students [64]. Pullmann and Allik [65] theorized that only contextual forms of self-esteem could be related to school performance compared to the general, unspecific, and broad concept ones. For this reason, they suggest dividing the self-esteem into two dimensions: general, associated with overall psychological wellbeing, and academic, defined as domain-specific self-esteem composed of cognitive elements and associated with behavioral outcomes as the school performance.

The idea that personality traits usually have a significant influence on the expression and management of emotions [66] is shared in the psychological literature. We were surprised by the lack of correlation between academic performance and emotional control. Despite the significant correlation with the average grade in the college study [61], in this sample, we did not find any direct relation between the reaction to failure and school performance. Hartley [67] suggests that it could be due to the college environment, which is more stressful and characterized by pressure compared to the high school one. Moreover, high school is compulsory in Italy, while a student who chooses to attend college can feel the pressure of showing it is a good one. Thus, to fail could be more oppressive for the college students compared to the high school ones who are more supported by their teachers to cope with stressful situations, having a more direct relationship with them.

All the subscales of the Relationship area (teachers, fellow students, parents) are correlated with school performance, coherently with a large number of previous studies (i.e., [68]). The relationship between teachers and students, however, is the more related one, consistently with the previous studies reported above [54,55,59]. This result, conversely, is different from the one we obtained in the college study, where this relationship correlated very slightly to the college performance. It could be explained by the different proximity and intensity of the relationship with schoolteachers compared to the college professors and for their role of “significant others” for the cognitive and affective development of their students.

Lastly, all the scales showed positive relationships with general and specific self-efficacy, one of the strongest predictors of scholastic performance. Further studies could deepen these relations by testing the presence of mediating effects, for instance, of H-Comp subscales between general self-efficacy and academic performance.

## 7. Conclusions

In this study, the H-Comp Scale showed to be potentially useful to assess high school students’ skills, attitudes and relationships for several reasons: it showed to possess excellent psychometric features; it allows us to measure, with a reasonable number of items, as many as twelve heterogeneous non-intellective students’ skills; and it is easy to administer even in larger test batteries. The H-Comp Scale is an agile scale, it is easy to complete (it takes about 15 min to respond to the whole scale), it can be administered by scholastic counselors and teachers, and it can be useful in helping them to recognize their students’ resources. 

**Practical implications.** The H-Comp Scale provides information on modifiable variables that can be used by psychologists, scholastic counselors, and teachers to support their students in increasing their scholastic performance. Used at the beginning of the new scholastic cycle, the H-Comp Scale allows one to identify students’ strength and weaknesses in approaching learning tasks, and to plan ad hoc intervention to prevent dropout risks. The relational area, furthermore, could provide useful information to students and teachers about the role that interpersonal relationships play in academic adjustment and performance. Allowing the identification of more critical dimensions, teachers, educators, and scholastic psychologists could implement specific training sessions to improve, for instance, their students’ self-efficacy and time management skills or to build more supportive relationships [69].

**Future studies**. The influence of non-intellective variables on both school and university performance should also be tested on primary school and middle-school students, adapting the questionnaire on these different targets. Both of our studies were conducted on Italian students; we hope to translate and validate the questionnaires in different languages and on students from different countries. Further studies could be addressed to verify the role of non-intellective competencies on wellbeing related variables, such as school satisfaction and life satisfaction, and their relationship with flourishing personality traits.

**Limitations**. Our research results must be read in light of several limitations.

Even if the questionnaire covers the most part of non-intellective competencies related to school performance with a brief number of items, many dimensions related to other non-intellective competence could not be included in the three areas of H-Comp. Therefore, when we built the C-Comp Scale, we conducted three focus groups with a different age group to identify the dimensions to consider in the questionnaire. Further studies are needed to confirm the validity of the scale in the high school student age group and to evaluate the role of other variables excluded by our instrument in school performance and school well-being. Possible bias could be related to the use of the cross-sectional design and self-reporting measures: both could have influenced the validity of the study in terms of reliability of the data, and they do not allow us to verify causal relationships. The sampling method could affect the possibility to extend our results to other contexts since our sample is not representative of the entire Italian population both for gender and types of school attended. If translated, cross-cultural studies are needed to confirm the factor invariance of the instrument for different samples of other cultural groups. Lastly, the H-Comp should be used on longitudinal studies to test, for instance, if their assessment at the beginning of a new student’s school path can affect their performance over time.

## Figures and Tables

**Table 1:** Areas’ structural validity: CFA goodness-of-fit indices of the Study, Self, and Relationships models for the pilot study.

Models	χ^2^ (*p*)	Df	RMSEA	CFI
Study	231.894 ***	98	0.061	0.960
Self	417.933 ***	160	0.067	0.920
Relationships	165.297 ***	51	0.079	0.912

Note. *** *p* < 0.001.

**Table 2:** Subscales’ psychometrics.

Subscales (Items)	*Mean*	*SD*	Skewness	Kurtosis
Intrinsic Motivation (4, 16, 28, 40)	10.55	2.663	0.200	0.376
Extrinsic Motivation (5, 17, 29, 41)	10.50	3.094	0.289	0.091
Time Management (8, 20, 32, 44)	11.29	3.014	0.254	0.054
Study Dedication (11, 23, 35, 47)	11.20	3.029	0.106	0.077
Learning Assessment (7, 19, 31, 43)	12.98	2.399	−0.016	0.467
Study Self-Efficacy (10, 22, 34, 46)	13.28	2.727	−0.069	0.472
Reaction to Failures (6, 18, 30, 42)	11.46	2.995	0.269	0.126
Emotional Control (12, 24, 36, 48)	11.22	3.096	0.249	0.149
General Self-Esteem (9, 21, 33, 45)	12.99	2.894	−0.090	0.089
Family Relationships (1, 13, 25, 37)	11.05	3.474	0.321	−0.298
Fellow Students Relationships (2, 14, 26, 38)	11.22	3.064	0.151	−0.177
Teachers Relationships (3, 15, 27, 39)	11.49	2.722	−0.022	0.159

**Table 3:** Measurement invariance levels for the Study model considering gender.

Model	χ^2^	df	CFI	RMSEA	Δχ^2^	Δdf	ΔCFI	ΔRMSEA
Configural invariance	479.575 ***	196	0.953	0.044				
Metric invariance	490.765 ***	208	0.954	0.043	11.190	12	0.001	0.001
Strong invariance	497.840 ***	218	0.954	0.042	7.075	10	0.000	0.001
Strict invariance	524.587 ***	234	0.952	0.041	26.747 *	16	0.002	0.001

Note. * *p* < 0.05; *** *p* < 0.001.

**Table 4:** Measurement invariance levels for the Self model considering gender.

Model	χ^2^	df	CFI	RMSEA	Δχ^2^	Δdf	ΔCFI	ΔRMSEA
Configural invariance	468.005 ***	196	0.927	0.044				
Metric invariance	482.816 ***	208	0.926	0.043	14.811	12	0.001	0.001
Strong invariance	498.368 ***	218	0.925	0.042	15.552	10	0.001	0.001
Strict invariance	542.338 ***	234	0.917	0.041	43.971 ***	16	0.008	0.001

Note. *** *p* < 0.001.

**Table 5:** Measurement invariance levels for the Relationships model considering gender.

Model	χ^2^	df	CFI	RMSEA	Δχ^2^	Δdf	ΔCFI	ΔRMSEA
Configural invariance	280.802 ***	102	0.925	0.049				
Metric invariance	303.966 ***	111	0.919	0.049	23.164 **	12	0.006	0.000
Strong invariance	315.711 ***	117	0.916	0.048	11.745	10	0.003	0.001
Strict invariance	350.169 ***	129	0.907	0.048	34.458 **	16	0.009	0.000

Note. ** *p* < 0.01; *** *p* < 0.001.

**Table 6:** Measurement invariance levels for the Study model considering grade.

Model	χ^2^	df	CFI	RMSEA	Δχ^2^	Δdf	ΔCFI	ΔRMSEA
Configural invariance	909.044 ***	490	0.934	0.034				
Metric invariance	964.098 ***	538	0.932	0.033	55.054	48	0.002	0.001
Strong invariance	1016.000 ***	578	0.931	0.032	51.902	40	0.001	0.001
Strict invariance	1119.830 ***	642	0.924	0.032	103.830 **	64	0.007	0.000

Note. ** *p* < 0.01; *** *p* < 0.001.

**Table 7:** Measurement invariance levels for the Self model considering grade.

Model	χ^2^	df	CFI	RMSEA	Δχ^2^	Δdf	ΔCFI	ΔRMSEA
Configural invariance	828.193 ***	490	0.914	0.031				
Metric invariance	913.102 ***	538	0.905	0.031	84.909 **	48	0.009	0.000
Strong invariance	966.753 ***	578	0.901	0.030	53.651	40	0.004	0.001
Strict invariance	1155.171 ***	642	0.869	0.095	188.418 ***	64	0.064	0.065

Note. ** *p* < 0.01; *** *p* < 0.001.

**Table 8:** Measurement invariance levels for the Relationships model considering grade.

Model	χ^2^	df	CFI	RMSEA	Δχ^2^	Δdf	ΔCFI	ΔRMSEA
Configural invariance	562.277 ***	336	0.909	0.030				
Metric invariance	578.466 ***	345	0.906	0.030	16.189	9	0.003	0.000
Strong invariance	589.580 ***	351	0.904	0.031	11.113	6	0.002	0.001
Strict invariance	612.651 ***	363	0.899	0.102	23.071 *	64	0.005	0.071

Note. * *p* < 0.05; *** *p* < 0.001.

**Table 9:** Areas’ structural validity: CFA goodness-of-fit-indices of Study, Self, and Relationships models for the main study.

Model	*N*	χ^2^ (*p*)	Df	RMSEA	CFI
Study	733	364.811 ***	98	0.061	0.957
Self	733	701.908 ***	160	0.068	0.903
Relationships	733	213.020 ***	51	0.066	0.933

Note. *** *p* < 0.001.

**Table 10:** Reliability: Cronbach’s alpha.

Subscales	Pilot Study (*n* = 364)	Main Study (*n* = 733)
Intrinsic Motivation	0.810	0.837
Extrinsic Motivation	0.820	0.778
Time Management	0.813	0.814
Study Dedication	0.890	0.875
Learning Assessment	0.717	0.695
Study Self-Efficacy	0.870	0.798
Reaction to Failures	0.824	0.778
Emotional Control	0.710	0.700
General Self-Esteem	0.802	0.798
Family Relationships	0.810	0.803
Fellow Students Relationships	0.751	0.730
Teachers Relationships	0.757	0.732

**Table 11:** Construct and Concurrent Validity: correlations between H-Comp subscales and different types of self-efficacy and scholastic performance.

	General Self-Efficacy	Scholastic Perceived Self-Efficacy	Social Perceived Self-Efficacy	Scholastic Performance
Intrinsic Motivation	0.202 ***	0.651 ***	0.110 ***	0.279 **
Extrinsic Motivation	0.211 ***	0.673 ***	0.120 **	0.365 **
Time Management	0.351 ***	0.708 ***	0.278 ***	0.343 **
Study Dedication	0.199 ***	0.714 ***	0.112 **	0.463 **
Learning Assessment	0.413 ***	0.457 ***	0.343 ***	0.205 **
Study Self-Efficacy	0.495 ***	0.621 ***	0.384 ***	0.440 **
Reaction to Failures	0.289 ***	0.151 ***	0.194 ***	0.057
Emotional Control	0.431 ***	0.191 ***	0.365 ***	−0.013
General Self-Esteem	0.614 ***	0.376 ***	0.572 ***	0.082 *
Family Relationships	0.060	0.397 ***	0.156 ***	0.199 **
Fellow Students Relationships	0.130 ***	0.232 ***	0.305 ***	0.129 **
Teachers Relationships	0.197 ***	0.477 ***	0.167 ***	0.234 **

Note. * *p* < 0.05; ** *p* < 0.01; *** *p* < 0.001.

## Data Availability

All data generated or analyzed during this study during the current study are not publicly available due to ethical reasons.

## Supplementary Materials

The following are available online at https://www.mdpi.com/article/10.3390/ejihpe11020041/s1, Table S1: 540 List of the Items in English with psychometrics.
