# Supplementary material for: The High School Competencies Scale (H-Comp Scale): A First Validation Study
Source: Eur J Investig Health Psychol Educ. 2021 Jun 17;11(2):570–84. doi: 10.3390/ejihpe11020041 (PMC8314347; doi:10.3390/ejihpe11020041)
Supplement: Supplementary file 1 [file ejihpe-11-00041-s001.zip › ejihpe-1235949-SI.pdf]

Scale	Number	Items	Mean	S.D.	Skewness	Kurtosis
Family Relationships	1	I keep my family members updated about my school performance	3,44	1,086	-0,150	-0,641
Fellow Students Relationships	2	I share, as much as possible, my doubts and my difficulties with my schoolmates	2,94	0,983	0,048	-0,412
Teachers Relationships	3	I think that almost all my teachers are available to dialogue	2,71	0,897	0,062	-0,208
Intrinsic Motivation	4	Most of the subjects that I study interest me	2,94	0,809	-0,018	-0,024
Extrinsic Motivation	5	Even on days when I am unmotivated, I manage to find the motivation in order to dedicate myself to my studies	2,41	1,042	0,398	-0,357
Reaction to Failures	6	I do not get discouraged by a lower grade than the one I thought I deserved	2,92	1,008	0,204	-0,290
Learning Assessment	7	I can estimate with a good degree of accuracy whether I am ready for a test or an oral exam or not	3,52	0,853	-0,162	-0,019
Time Management	8	I can plan my activities so as not to have an impact on my studies	3,02	1,000	0,054	-0,380
General Self-Esteem	9	I think I have good qualities in a lot of fields of my life	3,27	0,869	-0,084	0,139
Study Self-Efficacy	10	I think that I’m able to reach good results in my studies	3,55	0,821	-0,230	0,409
Study Dedication	11	I strive towards studying with a lot of commitment	2,80	0,895	0,126	0,033
Emotional Control	12	I do not have excessive anxiety problems when I have an oral test or a written one	2,72	1,146	0,217	-0,726
Family Relationships	13	I believe it is really important to involve my family members in what concerns my studies	2,71	1,092	0,228	-0,566
Fellow Students Relationships	14	When I need help, I ask my schoolmates	3,24	0,976	-0,123	-0,400
Teachers Relationships	15	In case I need it, I would not have difficulty asking my teachers for help	2,96	1,009	0,085	-0,420

Scale	Number	Items	Mean	S.D.	Skewness	Kurtosis
Intrinsic Motivation	16	In most cases I study willingly because I like to do that	2,24	0,890	0,380	-0,097
Extrinsic Motivation	17	I am always able to find a way in order to start studying even when I am not really interested	2,54	0,921	0,334	-0,040
Reaction to Failures	18	I do not get discouraged if I fail a written or an oral test	2,86	1,074	0,216	-0,561
Learning Assessment	19	While I am getting ready for a test or an oral exam, I am sufficiently aware of the learning level I reach as I progress	3,17	0,831	-0,025	0,447
Time Management	20	I am able to plan my workload in order not to be late	2,70	1,031	0,277	-0,325
General Self-Esteem	21	When I compare myself to the others, I realize I have good abilities	3,19	0,835	-0,111	0,392
Study Self-Efficacy	22	I consider myself a student with good study skills	3,16	0,842	-0,185	0,360
Study Dedication	23	I work hard in my studies to get good grades	3,11	0,955	0,038	-0,183
Emotional Control	24	Even if worked up, I can keep a clear head during a written or an oral test	3,08	1,027	0,014	-0,443
Family Relationships	25	I ask my family for help when I have difficulty with a subject	2,30	1,154	0,702	-0,252
Fellow Students Relationships	26	I was able to create a group of classmates to study with	2,44	1,215	0,475	-0,672
Teachers Relationships	27	I have good relationships with all teachers	3,05	0,966	-0,111	-0,160
Intrinsic Motivation	28	The books I am studying deal with topics that I like	2,62	0,794	-0,005	0,135
Extrinsic Motivation	29	Even on days when I am a bit unmotivated, I can find a way to study at least a little	2,57	0,944	0,434	0,126
Reaction to Failures	30	I do not get unmotivated because of my studying failures	2,79	1,012	0,208	-0,309

Scale	Number	Items	Mean	S.D.	Skewness	Kurtosis
Learning Assessment	31	Before a written or an oral test, I can predict quite precisely what grade I will get	2,79	0,934	-0,031	-0,131
Time Management	32	I can devote each subject enough time in order to get good results	2,76	0,810	0,181	0,200
General Self-Esteem	33	I think I am a person with good potential	3,52	0,878	-0,139	0,048
Study Self-Efficacy	34	I have the rights skills to be able to achieve good results in studying	3,58	0,861	-0,192	-0,032
Study Dedication	35	I am usually consistent in my studies	2,57	0,936	0,341	0,090
Emotional Control	36	I face oral or written tests calmly	2,79	1,081	0,154	-0,463
Family Relationships	37	I share my study difficulties with some of my family members	2,54	1,098	0,396	-0,479
Fellow Students Relationships	38	I often talk with my schoolmates about the best way to organize my studying activity	2,58	1,005	0,174	-0,514
Teachers Relationships	39	I think the teachers are helping me to develop my abilities	2,70	0,894	0,140	0,056
Intrinsic Motivation	40	Every subject teaches me something I am interested with	2,71	0,831	0,140	0,316
Extrinsic Motivation	41	I spend a lot of time on hobbies or friends provided that I have dedicated enough time to my studies	2,93	1,140	0,128	-0,744
Reaction to Failures	42	I do not get discouraged in front of difficulties that I encounter in my studies	2,83	0,903	0,137	-0,073
Learning Assessment	43	I can assess with some accuracy which subjects I am more or less prepared for	3,51	0,777	-0,159	0,375
Time Management	44	I can find the right balance between the time I use for studying and the time I use for leisure	2,80	1,027	0,241	-0,372
General Self-Esteem	45	I have high self-esteem	3,01	1,186	0,010	-0,775

Scale	Number	Items	Mean	S.D.	Skewness	Kurtosis
Study Self-Efficacy	46	I can state that I am a good student	2,98	0,904	-0,028	0,188
Study Dedication	47	I approach studying with great tenacity	2,70	0,860	0,025	0,156
Emotional Control	48	The idea of taking an oral or written test does not make me nervous	2,68	1,152	0,329	-0,623
